# Supplementary material for: Predicting rates of cognitive and functional decline in Alzheimer’s disease and mild cognitive impairment
Source: Commun Med (Lond). 2026 Feb 26;6:193. doi: 10.1038/s43856-026-01432-w (PMC13061986; doi:10.1038/s43856-026-01432-w)
Supplement: Supplementary file 2 — Supplementary Information [file 43856_2026_1432_MOESM2_ESM.pdf]

Supplementary Methods

Dementia diagnosis details

Participant diagnoses were retrieved from their electronic health records at baseline. Diagnoses were determined by clinicians, usually within community settings or memory clinics. Following an initial referral from the participant's GP to a community memory clinic, a comprehensive history is acquired from the patient and their family regarding changes in their behaviour, function, memory, and daily life, including the ability to carry out daily tasks. Psychological and cognitive assessments are usually performed alongside physical and neurological examinations and brain imaging. Collectively, this information is used to establish a suitable diagnosis, recorded in the patient's medical record, and formally documented in a clinic letter from the diagnosing physician. While processes vary slightly across clinicians, most will use the MMSE or MoCA for dementia diagnoses, and none will use the BADL. These diagnoses are the formally recorded diagnoses used at baseline in this study.

Throughout the course of the study, participants undergo further neuropsychiatric assessments and further brain imaging at regular intervals. An internal multi-disciplinary team of psychiatrists, radiologists, and neurologists discuss changes in individual diagnoses according to changes over time since baseline. If a change in diagnosis is agreed for the participant, this is recorded as the preferred diagnosis, overriding the baseline diagnosis established from the initial assessment in community memory clinics. This change is supported either by the natural progression of the disease (e.g. from MCI to Alzheimer's Disease) or as an alternative diagnosis due to an increase in evidence and thorough investigation that may not have been previously available.

Hyperparameter search spaces

Table S1: Hyperparameter tuning search spaces

Model	Hyperparameter	Search space
L1 Regularised Linear Regression	alpha	log-uniform[10^{-3}, 10]
	max_iter	[1000, 10000]
L2 Regularised Linear Regression	alpha	log-uniform[10^{-3}, 10^4]
ElasticNet Linear Regression	alpha	log-uniform[10^{-3}, 10]
	l1_ratio	uniform [0.1, 0.9]
XGBoost	n_estimators	50, 51, . . . , 500
	max_depth	2, 3, . . . , 10
	learning_rate	log-uniform[0.01, 0.1]
	min_child_weight	5, 6, . . . , 15
	subsample	uniform [0.6, 1.0]
	colsample_bytree	uniform [0.6, 1.0]
	gamma	log-uniform[0.1, 5]
	reg_alpha	log-uniform[0.1, 5]
	reg_lambda	log-uniform[1, 10]
Random Forest	n_estimators	50, 51, . . . , 500
	max_depth	2, 3, . . . , 10
Multilayer Perceptron	activation	['identity', 'logistic', 'tanh', 'relu']
	solver	['lbfgs', 'sgd', 'adam']
Gaussian Process	alpha	0.0001, 0.001, 0.01, 0.1, 1, 10
	kernel	*

*The Gaussian Process kernel search space included Radial Basis Function (RBF), DotProduct, and RationalQuadratic kernels. RBF length scale ω was sampled logarithmically over five values from 10^{-3} to 10. DotProduct ε_0 was sampled over the same 5 logarithmic values. For RationalQuadratic, both the length scale ω and scale-mixing parameter ϑ were sampled logarithmically from 10^{-3} and 10, generating 9 combinations (3×3) of these parameters.

Hyperparameters for the L1 and L2 regularised linear regression, ElasticNet linear regression, XGBoost, and Random Forest models were tuned using Bayesian optimisation, while hyperparameters for the MLP and GP regressors were tuned using a grid search. All searches were performed with 5-fold cross-validation. The MLP architecture consisted of three hidden layers: the first with dimensionality equal to the input size, the second with 75% of the previous layer's dimensions, and the third with 75% of the second's.

Extended model evaluation with neuroimaging features

To assess the added benefit of structural neuroimaging markers as features in our predictive models, a separate set of models were trained and evaluated using the ADNI dataset. One trained on the purely clinical features described in the main text, and one with structural MRI features added. For the neuroimaging + clinical model, 119 volumetric features derived from 1.5T structural MRI scans were included as inputs to our model of cognitive decline. MRI data was pre-processed and volumetric features generated based on previously published pipelines.[13] The model training and evaluation approach was consistent with the approach described in the main text of the paper. For both models, 608 trajectories were used.

Supplementary Results

Table S2: Individual clinical assessment counts for MMSE and BADL rate-of-change model cohorts

	MMSE Model	BADL Model
$n_{total\ assessments}$	316	296
n_{MMSE}	158	74
$n_{ADAS\text{-}Cog}$	79	74
n_{BADL}	79	148

MMSE: Mini-Mental State Exam, BADL: Bristol Activities of Daily Living questionnaire.

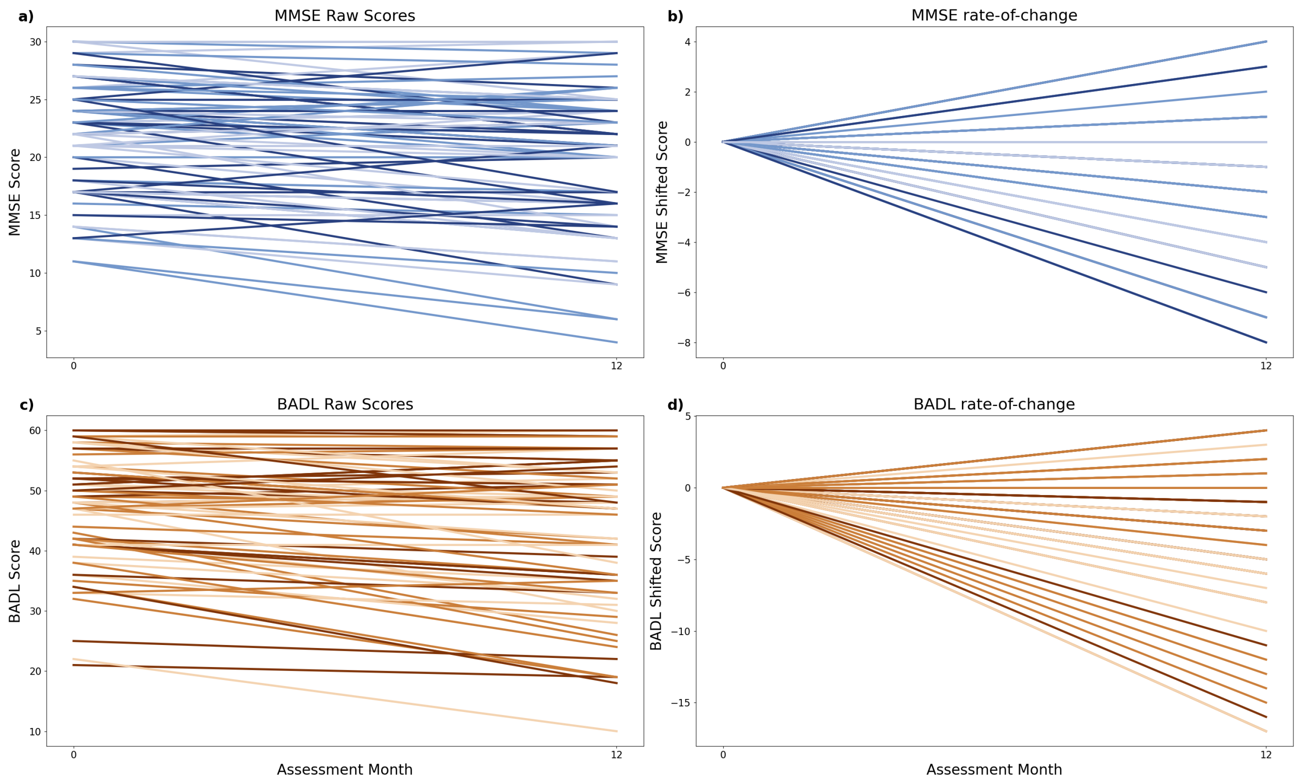

Figure S1: Heterogeneity in annual MMSE and BADL rates of change in the Minder cohort. Each individual trajectory is represented by a single line. a & c display raw 0- and 12-month assessment scores, in b & d, baseline scores are shifted to zero with 12-month scores shifted by the same amount to represent 12-month rate-of-change across study participants.

Table S3: Description of participants not included in modelling

	MMSE Model Cohort	BADL Model Cohort
Participants (n)	35	43
Female (%)	24 (69%)	28 (65%)
Age at baseline (SD)	82 (7)	83 (7)
Ethnicity		
White	22	24
Asian	1	1
Black	1	0
Other	1	2
N/A	10	16
Household		
PLWD lives alone	15	16
PLWD lives with partner	17	20
N/A	3	1
Primary diagnosis		
Alzheimer's Disease	30	40
Mild Cognitive Impairment	5	3
Baseline MMSE/BADL (min-max)	18.9 (2-30)	25.1 (0-58)

MMSE: Mini-Mental State Examination, BADL: Bristol Activities of Daily Living, PLWD: Person Living With Dementia

Table S4: Statistical association between baseline assessment scores for each BADL rate-of-change group

Assessment	Group 1	Group 2	p-value
Baseline ADAS-Cog	Slow Decline	Moderate Decline	0.0416
Baseline ADAS-Cog	Slow Decline	Steep Decline	0.0009
Baseline BADL	Slow Decline	Steep Decline	0.0138
Baseline MMSE	Slow Decline	Moderate Decline	0.0096
Baseline MMSE	Slow Decline	Steep Decline	0.0024

Model Features

Table S5: MMSE model features before and after feature selection with selected features' ElasticNet ϖ coe! cients

MMSE Model Features	Scale	ϖ
Year (Baseline MMSE)	0-1	
**Season (Baseline MMSE)**	**0-1**	**0.140**
**Month (Baseline MMSE)**	**0-1**	**0.140**
**Date (Baseline MMSE)**	**0-1**	**0.224**
**Day (Baseline MMSE)**	**0-1**	**0.119**
Country (Baseline MMSE)	0-1	
**County (Baseline MMSE)**	**0-1**	**0.964**
City (Baseline MMSE)	0-1	
Building (Baseline MMSE)	0-1	
Floor (Baseline MMSE)	0-1	
**Registration (Baseline MMSE)**	**0-3**	**0.153**
World (Baseline MMSE)	0-5	
**Recall (Baseline MMSE)**	**0-3**	**0.311**
**Naming (Baseline MMSE)**	**0-2**	**0.253**
Repeat (Baseline MMSE)	0-1	
Closeyoureyes (Baseline MMSE)	0-1	
Writesentence (Baseline MMSE)	0-1	
**Copy pentagons (Baseline MMSE)**	**0-1**	**0.521**
Commands (Baseline MMSE)	0-3	
**Baseline MMSE**	**0-30**	**1.190**
**Spoken language (Baseline ADAS)**	**0-10**	**0.419**
Comprehension language (Baseline ADAS)	0-5	
Word finding (Baseline ADAS)	0-5	
Remembering instructions (Baseline ADAS)	0-5	
**Word recall (Baseline ADAS)**	**0-5**	**0.551**
**Naming (Baseline ADAS)**	**0-8**	**0.106**
Commands (Baseline ADAS)	0-12	
Constructional praxis (Baseline ADAS)	0-5	
**Ideational praxis (Baseline ADAS)**	**0-5**	**1.172**
**Orientation (Baseline ADAS)**	**0-5**	**0.201**
**Word recognition (Baseline ADAS)**	**0-10**	**0.213**
**Baseline ADAS-Cog**	**0-70**	**0.794**
Sex	1=M, 2=F	
Age	integer	
Age-Sex Intersection	integer	

Selected features are indicated in bold.

Table S6: MMSE model features including comorbidities before and after feature selection with selected features' ElasticNet ϖ coe! cients

MMSE Model Features	Scale	ϖ
Year (Baseline MMSE)	0-1	
**Season (Baseline MMSE)**	**0-1**	**0.088**
**Month (Baseline MMSE)**	**0-1**	**0.065**
**Date (Baseline MMSE)**	**0-1**	**0.172**
**Day (Baseline MMSE)**	**0-1**	**0.098**
Country (Baseline MMSE)	0-1	
**County (Baseline MMSE)**	**0-1**	**0.866**
City (Baseline MMSE)	0-1	
Building (Baseline MMSE)	0-1	
**Floor (Baseline MMSE)**	**0-1**	**0.133**
**Registration (Baseline MMSE)**	**0-3**	**0.161**
World (Baseline MMSE)	0-5	
**Recall (Baseline MMSE)**	**0-3**	**0.301**
**Naming (Baseline MMSE)**	**0-2**	**0.137**
Repeat (Baseline MMSE)	0-1	
Closeyoureyes (Baseline MMSE)	0-1	
Writesentence (Baseline MMSE)	0-1	
**Copy pentagons (Baseline MMSE)**	**0-1**	**0.458**
Commands (Baseline MMSE)	0-3	
**Baseline MMSE**	**0-30**	**1.191**
**Spoken language (Baseline ADAS)**	**0-10**	**0.365**
Comprehension language (Baseline ADAS)	0-5	
**Word finding (Baseline ADAS)**	**0-5**	**0.071**
Remembering instructions (Baseline ADAS)	0-5	
**Word recall (Baseline ADAS)**	**0-5**	**0.532**
**Naming (Baseline ADAS)**	**0-8**	**0.102**
Commands (Baseline ADAS)	0-12	
Constructional praxis (Baseline ADAS)	0-5	
**Ideational praxis (Baseline ADAS)**	**0-5**	**1.051**
**Orientation (Baseline ADAS)**	**0-5**	**0.298**
**Word recognition (Baseline ADAS)**	**0-10**	**0.183**
**Baseline ADAS-Cog**	**0-70**	**0.811**
Sex	1= M, 2= F	
Age	integer	
Age-Sex Intersection	integer	
**I: Certain infectious and parasitic diseases**	**binary**	**0.146**
II: Neoplasms	binary	
III: Diseases of the blood and blood-forming organs and certain disorders involving the immune mechanism	binary	
IV: Endocrine, nutritional and metabolic diseases	binary	
V: Mental and behavioural disorders	binary	
VI: Diseases of the nervous system	binary	
VII: Diseases of the eye and adnexa	binary	
VIII: Diseases of the ear and mastoid process	binary	
**IX: Diseases of the circulatory system**	**binary**	**-0.046**
X: Diseases of the respiratory system	binary	
**XI: Diseases of the digestive system**	**binary**	**0.047**
XII: Diseases of the skin and subcutaneous tissue	binary	
XIII: Diseases of the musculoskeletal system and connective tissue	binary	
XIV: Diseases of the genitourinary system	binary	
XV-XXII: Other comorbid conditions	binary	

Selected features are indicated in bold.

Table S7: BADL model features before and after feature selection with selected features' ElasticNet ϖ coe! cients

BADL Model Features	Scale	ϖ
Food score (Baseline BADL)	0-3	1.244
Eating score (Baseline BADL)	0-3	
Drink score (Baseline BADL)	0-3	1.135
Drinking score (Baseline BADL)	0-3	
Dressing score (Baseline BADL)	0-3	1.005
Hygiene score (Baseline BADL)	0-3	0.752
Teeth score (Baseline BADL)	0-3	0.480
Bath shower score (Baseline BADL)	0-3	
Toilet commode score (Baseline BADL)	0-3	
Transfers score (Baseline BADL)	0-3	0.583
Mobility score (Baseline BADL)	0-3	
Orientation time score (Baseline BADL)	0-3	0.680
Orientation space score (Baseline BADL)	0-3	
Communication score (Baseline BADL)	0-3	
Telephone score (Baseline BADL)	0-3	
Housework gardening score (Baseline BADL)	0-3	0.672
Shopping score (Baseline BADL)	0-3	0.869
Finances score (Baseline BADL)	0-3	1.338
Games hobbies score (Baseline BADL)	0-3	0.915
Transport score (Baseline BADL)	0-3	0.678
Baseline BADL	0-60	1.155
Spoken language (Baseline ADAS)	0-10	
Comprehension language (Baseline ADAS)	0-5	
Word finding (Baseline ADAS)	0-5	0.725
Remembering instructions (Baseline ADAS)	0-5	
Word recall (Baseline ADAS)	0-5	0.884
Naming (Baseline ADAS)	0-8	
Commands (Baseline ADAS)	0-12	
Constructional praxis (Baseline ADAS)	0-5	
Ideational praxis (Baseline ADAS)	0-5	
Orientation (Baseline ADAS)	0-5	
Word recognition (Baseline ADAS)	0-10	0.781
Baseline ADAS-Cog	0-70	0.784
Sex	1= M, 2= F	
Age	integer	-1.050
Age-Sex Intersection	integer	

Selected features are indicated in bold.

Performance of all assessed models

Table S8: Model performance for all models of 12-month cognitive decline

Comorbidities	Model	MSE (95% CI)	MAE (95% CI)	R^2 (95% CI)
Excluded	L1 Regularised Linear Regression	7.84 (6.15 – 9.52)	2.28 (1.98 – 2.58)	0.69 (0.57 – 0.80)
	L2 Regularised Linear Regression	9.51 (7.49 – 11.54)	2.50 (2.16 – 2.83)	0.61 (0.43 – 0.78)
	ElasticNet Linear Regression	8.01 (6.61 – 9.42)	2.29 (2.05 – 2.54)	0.68 (0.56 – 0.80)
	XGBoost	9.06 (7.20 – 10.92)	2.34 (2.07 – 2.61)	0.62 (0.43 – 0.81)
	Random Forest	9.07 (7.32 – 10.82)	2.43 (2.16 – 2.69)	0.63 (0.48 – 0.78)
	Multilayer Perceptron	16.52 (13.57 – 19.46)	3.28 (2.98 – 3.58)	0.35 (0.12 – 0.58)
	Gaussian Process	9.28 (5.16 – 13.40)	2.45 (1.90 – 3.00)	0.64 (0.42 – 0.85)
Included	L1 Regularised Linear Regression	7.83 (6.21 – 9.44)	2.28 (1.99 – 2.56)	0.69 (0.58 – 0.80)
	L2 Regularised Linear Regression	10.21 (7.59 – 12.83)	2.53 (2.15 – 2.90)	0.64 (0.55 – 0.72)
	ElasticNet Linear Regression	8.03 (6.52 – 9.54)	2.29 (2.04 – 2.54)	0.68 (0.56 – 0.80)
	XGBoost	9.38 (7.39 – 11.37)	2.44 (2.14 – 2.73)	0.62 (0.46 – 0.78)
	Random Forest	8.83 (7.15 – 10.50)	2.40 (2.14 – 2.67)	0.64 (0.49 – 0.79)
	Multilayer Perceptron	21.46 (14.54 – 28.37)	3.59 (2.93 – 4.24)	0.15 (-0.25 – 0.56)
	Gaussian Process	9.86 (6.04 – 13.67)	2.53 (2.03 – 3.04)	0.63 (0.43 – 0.82)

MSE: Mean-squared-error, MAE: Mean-absolute-error, MMSE: Mini-Mental State Exam, BADL: Bristol Activities of Daily Living Questionnaire.

Table S9: Model performance for all models of 12-month cognitive decline, BADL ablation study

Comorbidities	Model	MSE (95% CI)	MAE (95% CI)	R^2 (95% CI)
Excluded	L1 Regularised Linear Regression	5.75 (4.58 – 6.92)	1.90 (1.70 – 2.09)	0.73 (0.66 – 0.80)
	L2 Regularised Linear Regression	7.67 (6.03 – 9.31)	2.27 (2.06 – 2.47)	0.64 (0.56 – 0.73)
	ElasticNet Linear Regression	5.64 (4.40 – 6.88)	1.85 (1.65 – 2.04)	0.74 (0.67 – 0.81)
	XGBoost	7.24 (5.36 – 9.11)	2.10 (1.78 – 2.42)	0.64 (0.50 – 0.78)
	Random Forest	7.69 (5.82 – 9.55)	2.19 (1.94 – 2.44)	0.63 (0.50 – 0.76)
	Multilayer Perceptron	19.00 (13.22 – 24.78)	3.41 (2.94 – 3.87)	0.14 (-0.09 – 0.38)
	Gaussian Process	7.14 (6.17 – 8.12)	2.19 (2.00 – 2.37)	0.66 (0.58 – 0.74)
Included	L1 Regularised Linear Regression	5.66 (4.54 – 6.79)	1.88 (1.66 – 2.09)	0.73 (0.66 – 0.80)
	L2 Regularised Linear Regression	7.64 (6.08 – 9.21)	2.25 (2.00 – 2.50)	0.64 (0.54 – 0.74)
	ElasticNet Linear Regression	5.60 (4.42 – 6.78)	1.84 (1.64 – 2.04)	0.74 (0.67 – 0.80)
	XGBoost	7.69 (5.84 – 9.53)	2.16 (1.87 – 2.44)	0.62 (0.49 – 0.76)
	Random Forest	7.62 (5.91 – 9.33)	2.20 (1.96 – 2.45)	0.63 (0.52 – 0.75)
	Multilayer Perceptron	16.38 (14.49 – 18.27)	3.40 (3.21 – 3.59)	0.20 (-0.05 – 0.46)
	Gaussian Process	8.10 (5.92 – 10.28)	2.22 (1.99 – 2.46)	0.63 (0.53 – 0.73)

MSE: Mean-squared-error, MAE: Mean-absolute-error, MMSE: Mini-Mental State Exam, BADL: Bristol Activities of Daily Living Questionnaire.

Table S10: Model performance for all models of 12-month functional decline

Comorbidities	Model	MSE (95% CI)	MAE (95% CI)	R^2 (95% CI)
Excluded	L1 Regularised Linear Regression	25.72 (20.22 – 31.22)	4.08 (3.69 – 4.46)	0.74 (0.70 – 0.79)
	L2 Regularised Linear Regression	25.72 (20.22 – 31.22)	4.08 (3.69 – 4.46)	0.74 (0.70 – 0.79)
	ElasticNet Linear Regression	23.96 (18.69 – 29.23)	3.93 (3.52 – 4.35)	0.76 (0.71 – 0.81)
	XGBoost	32.50 (26.08 – 38.93)	4.57 (4.06 – 5.09)	0.67 (0.59 – 0.74)
	Random Forest	28.82 (22.39 – 35.24)	4.30 (3.90 – 4.70)	0.71 (0.64 – 0.77)
	Multilayer Perceptron	68.57 (54.85 – 82.30)	6.51 (5.84 – 7.19)	0.30 (0.14 – 0.45)
	Gaussian Process	34.37 (27.84 – 40.90)	4.80 (4.34 – 5.26)	0.65 (0.56 – 0.73)
Included	L1 Regularised Linear Regression	28.16 (22.08 – 34.24)	4.32 (3.87 – 4.77)	0.72 (0.69 – 0.76)
	L2 Regularised Linear Regression	30.11 (23.94 – 36.28)	4.28 (3.74 – 4.83)	0.70 (0.66 – 0.75)
	ElasticNet Linear Regression	26.64 (20.81 – 32.47)	4.11 (3.64 – 4.58)	0.74 (0.69 – 0.78)
	XGBoost	34.99 (29.28 – 40.71)	4.77 (4.31 – 5.24)	0.64 (0.57 – 0.71)
	Random Forest	27.97 (20.68 – 35.26)	4.22 (3.78 – 4.66)	0.72 (0.66 – 0.78)
	Multilayer Perceptron	52.20 (46.11 – 58.28)	5.89 (5.29 – 6.49)	0.46 (0.37 – 0.55)
	Gaussian Process	36.41 (28.86 – 43.96)	4.94 (4.31 – 5.57)	0.64 (0.56 – 0.71)

MSE: Mean-squared-error, MAE: Mean-absolute-error, MMSE: Mini-Mental State Exam, BADL: Bristol Activities of Daily Living Questionnaire.

Table S11: Model performance for all models of 12-month functional decline, cognitive assessment ablation studies

Excluded	Model	MSE (95% CI)	MAE (95% CI)	R^2 (95% CI)
MMSE	L1 Regularised Linear Regression	25.16 (20.00 – 30.32)	4.06 (3.69 – 4.43)	0.75 (0.69 – 0.80)
	L2 Regularised Linear Regression	27.22 (21.64 – 32.80)	4.32 (3.86 – 4.78)	0.72 (0.67 – 0.78)
	ElasticNet Linear Regression	22.93 (17.87 – 28.00)	3.88 (3.46 – 4.30)	0.77 (0.72 – 0.82)
	XGBoost	32.28 (26.08 – 38.47)	4.51 (4.06 – 4.95)	0.67 (0.60 – 0.74)
	Random Forest	29.13 (23.36 – 34.90)	4.24 (3.84 – 4.64)	0.70 (0.63 – 0.77)
	Multilayer Perceptron	52.71 (39.87 – 65.56)	5.54 (4.81 – 6.28)	0.45 (0.29 – 0.62)
	Gaussian Process	30.02 (25.06 – 34.98)	4.63 (4.27 – 4.99)	0.70 (0.65 – 0.74)
ADAS-Cog	L1 Regularised Linear Regression	24.79 (18.84 – 30.74)	3.92 (3.46 – 4.38)	0.76 (0.71 – 0.80)
	L2 Regularised Linear Regression	27.11 (21.12 – 33.10)	4.09 (3.62 – 4.56)	0.73 (0.67 – 0.78)
	ElasticNet Linear Regression	24.38 (19.48 – 29.29)	3.94 (3.55 – 4.33)	0.75 (0.71 – 0.80)
	XGBoost	34.88 (26.87 – 42.89)	4.92 (4.35 – 5.50)	0.63 (0.52 – 0.74)
	Random Forest	29.12 (23.28 – 34.97)	4.36 (3.91 – 4.81)	0.70 (0.62 – 0.78)
	Multilayer Perceptron	62.26 (47.74 – 76.78)	6.22 (5.56 – 6.89)	0.38 (0.26 – 0.49)
	Gaussian Process	35.26 (27.96 – 42.56)	4.63 (4.16 – 5.11)	0.63 (0.53 – 0.73)
MMSE & ADAS-Cog	L1 Regularised Linear Regression	27.75 (21.01 – 34.49)	4.16 (3.72 – 4.59)	0.72 (0.66 – 0.78)
	L2 Regularised Linear Regression	27.57 (20.21 – 34.92)	4.23 (3.77 – 4.70)	0.73 (0.67 – 0.78)
	ElasticNet Linear Regression	25.45 (18.47 – 32.42)	3.99 (3.54 – 4.45)	0.75 (0.69 – 0.80)
	XGBoost	31.36 (23.85 – 38.87)	4.53 (4.05 – 5.00)	0.68 (0.60 – 0.76)
	Random Forest	31.13 (24.86 – 37.40)	4.29 (3.85 – 4.74)	0.68 (0.59 – 0.76)
	Multilayer Perceptron	66.50 (50.43 – 82.57)	6.40 (5.53 – 7.28)	0.33 (0.16 – 0.50)
	Gaussian Process	26.69 (22.68 – 30.71)	4.11 (3.81 – 4.42)	0.73 (0.68 – 0.77)

MSE: Mean-squared-error, MAE: Mean-absolute-error, MMSE: Mini-Mental State Exam, BADL: Bristol Activities of Daily Living Questionnaire.

Predictive model performance with reduced features

We conducted a reverse feature selection for the prediction models within the criteria that meet the guidelines introduced in Riley et al.[39] The results demonstrated that 13 features were required for the MMSE model and 14 features were required for the BADL model to make robust predictions and meet the sample size criteria. Tables below summarise the performance of the predictive models with the adjusted dimensions.

Table S12: Model performance for models of 12-month cognitive decline, 13 features selected.

Comorbidities	Model	MSE (95% CI)	MAE (95% CI)	R^2 (95% CI)
Excluded	L1 Regularised Linear Regression	5.73 (4.53 – 6.94)	1.89 (1.69 – 2.10)	0.73 (0.66 – 0.80)
	L2 Regularised Linear Regression	7.73 (5.71 – 9.75)	2.23 (1.98 – 2.48)	0.64 (0.55 – 0.74)
	ElasticNet	5.60 (4.38 – 6.82)	1.85 (1.65 – 2.05)	0.74 (0.67 – 0.81)
	XGBoost	7.17 (5.22 – 9.11)	2.08 (1.75 – 2.41)	0.65 (0.51 – 0.78)
	Random Forest	7.55 (5.84 – 9.25)	2.17 (1.92 – 2.43)	0.63 (0.51 – 0.75)
Included	L1 Regularised Linear Regression	5.75 (4.60 – 6.90)	1.89 (1.69 – 2.10)	0.73 (0.65 – 0.80)
	L2 Regularised Linear Regression	8.97 (7.07 – 10.88)	2.36 (2.13 – 2.59)	0.57 (0.43 – 0.70)
	ElasticNet	5.64 (4.43 – 6.84)	1.85 (1.65 – 2.05)	0.74 (0.67 – 0.80)
	XGBoost	7.51 (5.84 – 9.19)	2.15 (1.89 – 2.41)	0.63 (0.51 – 0.76)
	Random Forest	7.58 (5.67 – 9.48)	2.17 (1.91 – 2.43)	0.64 (0.51 – 0.76)

MSE: Mean-squared-error, MAE: Mean-absolute-error.

Table S13: Model performance for models of 12-month functional decline, 14 features selected.

Model	MSE (95% CI)	MAE (95% CI)	R^2 (95% CI)
L1 Regularised Linear Regression	25.36 (20.01 – 30.70)	4.08 (3.70 – 4.46)	0.74 (0.69 – 0.80)
L2 Regularised Linear Regression	29.51 (23.34 – 35.68)	4.43 (3.96 – 4.91)	0.70 (0.65 – 0.76)
ElasticNet	24.42 (19.19 – 29.65)	4.08 (3.66 – 4.50)	0.75 (0.70 – 0.80)
XGBoost	31.53 (24.50 – 38.56)	4.37 (3.87 – 4.86)	0.68 (0.60 – 0.75)
Random Forest	28.89 (22.44 – 35.33)	4.20 (3.76 – 4.63)	0.71 (0.65 – 0.77)

MSE: Mean-squared-error, MAE: Mean-absolute-error.

Extended model evaluation with neuroimaging features

Models of cognitive decline trained using only clinical features and those that also included neuroimaging features demonstrated comparable performance (Table S14). Overall, these results suggest that inclusion of volumetric neuroimaging features do not meaningfully improve the performance or reliability of predictive models of 12-month cognitive decline in the ADNI cohort.

Table S14: Model performance for models of 12-month cognitive decline, with and without neuroimaging features in the ADNI cohort.

Neuroimaging	Model	MSE (95% CI)	MAE (95% CI)	R^2 (95% CI)
Included	L1 Regularised Linear Regression	7.09 (6.04 – 8.14)	2.02 (1.88 – 2.16)	0.71 (0.66 – 0.76)
	L2 Regularised Linear Regression	7.66 (6.42 – 8.91)	2.13 (1.99 – 2.27)	0.69 (0.63 – 0.75)
	ElasticNet	7.16 (6.08 – 8.24)	2.02 (1.90 – 2.14)	0.71 (0.66 – 0.76)
Excluded	L1 Regularised Linear Regression	7.16 (6.08 – 8.24)	2.04 (1.90 – 2.18)	0.71 (0.66 – 0.76)
	L2 Regularised Linear Regression	7.47 (6.37 – 8.57)	2.09 (1.96 – 2.23)	0.70 (0.64 – 0.75)
	ElasticNet	7.18 (6.13 – 8.24)	2.04 (1.91 – 2.17)	0.71 (0.66 – 0.76)

MSE: Mean-squared-error, MAE: Mean-absolute-error.

Data ablation study

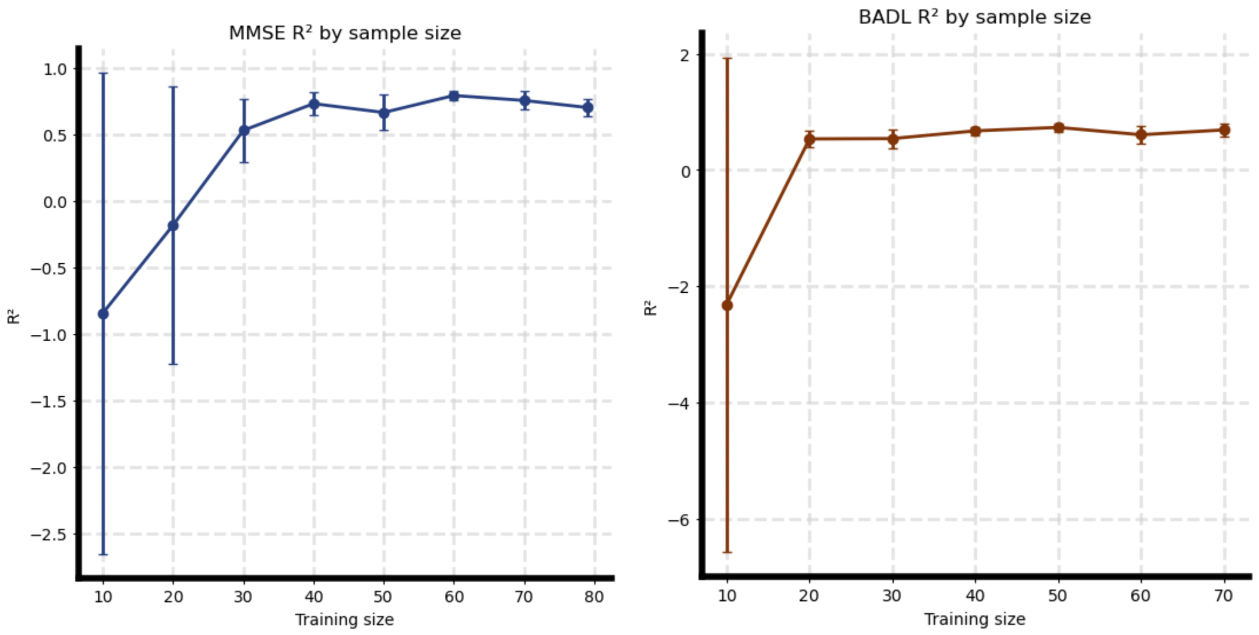

(a) (b)

Figure S2: Data ablation study results. R^2 for models trained to predict MMSE (a) and BADL (b) scores using 10-80 samples. Error bars represent 95% confidence intervals.
