# Supplementary material for: Predicting rates of cognitive and functional decline in Alzheimer’s disease and mild cognitive impairment
Source: Commun Med (Lond). 2026 Feb 26;6:193. doi: 10.1038/s43856-026-01432-w (PMC13061986; doi:10.1038/s43856-026-01432-w)
Supplement: Supplementary file 3 — Description of Additional Supplementary Files [file 43856_2026_1432_MOESM3_ESM.pdf]

# Description of Additional Supplementary Files

**File name:** Supplementary Data 1

**Description:** Source Data for Figure 2

**File name:** Supplementary Data 2

**Description:** Source Data for Figure 3
